# Recovery of Metals from Acid Mine Drainage by Bioelectrochemical System Inoculated with a Novel Exoelectrogen, *Pseudomonas* sp. E8

**DOI:** 10.3390/microorganisms8010041

**Published:** 2019-12-24

**Authors:** Chenbing Ai, Shanshan Hou, Zhang Yan, Xiaoya Zheng, Charles Amanze, Liyuan Chai, Guanzhou Qiu, Weimin Zeng

**Affiliations:** 1 School of Metallurgy and Environment, Central South University, Changsha 410083, Hunan, Chinachailiyuan@csu.edu.cn (L.C.); 2 School of Minerals Processing and Bioengineering, Central South University, Changsha 410083, Hunan, China; 185611026@csu.edu.cn (S.H.); 201910107647@mail.scut.edu.cn (Z.Y.); zhengxiaoya@csu.edu.cn (X.Z.); charles.amanze@csu.edu.cn (C.A.); qgz@csu.edu.cn (G.Q.); 3 Chinese National Engineering Research Center for Control and Treatment of Heavy Metal Pollution, Central South University, Changsha 410083, Hunan, China; 4 Key Laboratory of Biometallurgy of Ministry of Education, Central South University, Changsha 410083, Hunan, China; 5 College of Environmental Science and Engineering, Fujian Key Laboratory of Pollution Control & Resource Reuse, Fujian Normal University, Fuzhou 350007, Fujian, China

**Keywords:** acid mine drainage, metal recovery, exoelectrogen, microbial fuel cell, microbial electrolysis cell

## Abstract

Acid mine drainage (AMD) is a typical source of environmental pollution ascribing to its characteristics of high acidity and heavy metal content. Currently, most strategies for AMD treatment merely focus on metal removal rather than metal recovery. However, bioelectrochemical system (BES) is a promising technology to simultaneously remove and recover metal ions from AMD. In this study, both cupric ion and cadmium ion in simulated AMD were effectively recovered by BES inoculated with a novel exoelectrogen, *Pseudomonas* sp. E8, that was first isolated from the anodic electroactive biofilm of a microbial fuel cell (MFC) in this study. *Pseudomonas* sp. E8 is a facultative anaerobic bacterium with a rod shape, 0.43–0.47 μm wide, and 1.10–1.30 μm long. *Pseudomonas* sp. E8 can agglomerate on the anode surface to form a biofilm in the single-chamber MFC using diluted Luria-Bertani (LB) medium as an energy substrate. A single-chamber MFC containing the electroactive *Pseudomonas* sp. E8 biofilms has a maximum output voltage of 191 mV and a maximum power density of 70.40 mW/m^2^, which is much higher than those obtained by most other exoelectrogenic strains in the genus of *Pseudomonas*. Almost all the Cu^2+^ (99.95% ± 0.09%) and Cd^2+^ (99.86% ± 0.04%) in simulated AMD were selectively recovered by a microbial fuel cell (MFC) and a microbial electrolysis cell (MEC). After the treatment with BES, the high concentrations of Cu^2+^(184.78 mg/L), Cd^2+^(132.25 mg/L), and total iron (49.87 mg/L) in simulated AMD were decreased to 0.02, 0.19, and 0 mg/L, respectively. Scanning electron micrograph (SEM), energy dispersive X-ray spectrometry (EDXS) and X-ray diffraction (XRD) analysis indicate that the Cu^2+^ and Cd^2+^ in simulated AMD were selectively recovered by microbial electrochemical reduction as Cu^0^ (together with trace amounts of Cu_2_O) or Cd^0^ on the cathode surface. Collectively, data suggest that *Pseudomonas* sp. E8 has great potential for AMD treatment and metal recovery.

## 1. Introduction

Acid mine drainage (AMD) is a metal-rich acidic water occurring in abandoned, inactive, and active mining sites, around open-pit mining operations, and mine waste sites, which poses significant environmental and economic challenges to the mining industry [1]. AMD is primarily generated from multi-step oxidation processes when the mineral-containing sulfides are exposed to air and water, especially in the presence of chemolithotrophic acidophiles [2,3]. The chemical reactions that occur in AMD are well understood processes and have been described in detail in many previous studies [4,5,6]. There are a tremendous amount of abandoned mines and actively operating mines that generate huge tons of AMD around the world, especially in China and Australia [7]. Around 15,000 m^3^/day of AMD is produced from the Liwu mud-retaining dam located in a mining region in northern Guangdong, China [8]. Similarly, Guo et al. [9] reported that the Dexing Copper Mine located in Dexing city, Jiangxi province, China, discharged more than 4 × 10^4^ tons of acidic mine wastewater (AMD) everyday, which seriously polluted the soil and various natural water sources downstream of this mine. Studies have shown that AMD tends to penetrate into soil and has long-term negative effects on plant growth and human health [10,11]. In order to maintain the environmental sustainability regarding mining activities, effective and efficient technologies that can tackle the remediation of AMD are highly required.

The AMD remediation technologies can be divided into active and passive treatment. Active treatments are mainly used to achieve the discharge of standard wastewater by adjusting pH, including fractional precipitation, selective precipitation by electrochemical reaction, and so on [12]. Passive treatments usually achieve precipitation by creating reduction conditions, using organic matter as alkaline agents to achieve sulfide metal precipitation, which include aerobic wetlands, composting reactors, and so on [13]. However, these technologies require the continuous supply of a large quantity of chemicals and energy, and a high processing cost [14]. In addition, those technologies have the drawbacks of either low remediation efficiency or producing new wastes (e.g., sludge, brines, and spent media) which require further treatment. Most of these remediation strategies focus on removal of metals mainly by precipitation, whereas only a few researchers focus on the recovery of valuable metals. However, a bioelectrochemical system (BES) is a promising water-treatment technology to simultaneously remove and recover metal ions from AMD [15].

A bioelectrochemical system is an appealing microbial technology that treats various organic wastewater together with organic/inorganic pollutants and simultaneously converts chemical energy into electrical energy through the biocatalysis of exoelectrogen [16]. According to the difference regarding configuration and function, BES can be mainly divided into the following four categories: microbial fuel cell (MFC), microbial electrolysis cell (MEC), microbial desalinating cell (MDC), microbial electrosynthesis cell (MES) [17]. BES has received extensive studies at bench-scale or at the pilot level, both in theoretical research and process development, due to its characteristics of high capacities for wastewater treatment and effective energy generation [17,18]. However, a bioelectrochemical system has not been successfully applied to the treatment of actual wastewater yet, which was mainly attributed to the low power density. Since the exoelectrogen plays a pivotal role for the performance of BES, therefore, it is feasible to improve the power density of BES by inoculating with an effective exoelectrogen.

In this study, a novel exoelectrogen *Pseudomonas* sp. E8 was first isolated from the anodic bioelectroactive biofilm of MFC. The electrochemical properties (e.g., maximum output voltage, power density, polarization curve, and coulombic efficiency) of *Pseudomonas* sp. E8 were characterized with fuel cell techniques and compared with those of other exoelectrogenic strains in the genus of *Pseudomonas*. The feasibility of using a bioelectrochemical system with *Pseudomonas* sp. E8 to treat simulated AMD containing high concentrations of Cu^2+^, Cd^2+^, and Fe^3+^ was evaluated. In addition, the mechanism for ion removal on the surface of the cathode was explored. Data indicate that *Pseudomonas* sp. E8 has great potential for AMD treatment and metal recovery.

## 2. Materials and Methods

### 2.1. Isolation and Identification of Pseudomonas sp. E8

The novel exoelectrogenic strain used in this study was isolated from the anode of a microbial fuel cell (MFC), which was inoculated with the activated sludge from Xinkaipu Municipal Sewage Treatment Plant (Changsha, China) and fed with 20 mM sodium acetate. A wisp of anode was transferred to a 2 mL centrifuge tube containing 20 mM phosphate buffer and vibrated for 1 min to separate microbial cells from the electrode. Colonies with various shapes and sizes were formed by streaking the suspension on the Luria-Bertani (LB) plate. The hexagonally structured electrochromic WO_3_ nanomaterials were used to screen electrogenic strains from these colonies [19]. An efficient electrogenic strain with the remarkable capacity to color electrochromic WO_3_ nanomaterials was obtained. The genomic DNA of this strain was extracted using the TIANamp Bacteria DNA Kit (Tiangen, Beijing, China) following the supplier’s instructions. The 16S rRNA gene of this strain was amplified and sequenced using the universal primer pair 27F (5’-AGAGTTTGATCCTGGCTCAG-3’) and 1492R (5’-GGTTACCTTGTTACGACTT-3’). For identification of the isolate, the amplified 16S rRNA gene sequence was blasted with full sequences available in the NCBI GenBank database (using BLASTP). Based on the blasted results of the 16S rDNA gene sequence, this novel exoelectrogenic strain was named as *Pseudomonas* sp. E8. The 16S rRNA gene sequence of this strain was deposited in NCBI with the accession number MN589671. The phylogenetic analysis of *Pseudomonas* sp. E8 was carried out using MEGA-7.0 software by the neighbor-joining method with 1000 bootstrap [20].

### 2.2. Configuration of MFC Reactors and MEC Reactors

A cubical single-chamber MFC reactor was constructed to evaluate the electrochemical properties of *Pseudomonas* sp. E8. The single-chamber MFC reactor with a cylindrical chamber (3 cm in diameter × 4 cm in length) was made of polymethyl methacrylate (PMMA). Each MFC reactor (with a working volume of 28 mL) consisted of a carbon brush (1.5 cm in radius × 3 cm in length) as an anode and a carbon cloth (projected surface area of 7.07 cm^2^) as a cathode. The anode and cathode were connected with an external resistance of 1000 Ω by titanium wire. In order to remove contaminants on the surface, both the carbon brush and carbon cloth were soaked overnight in acetone, followed by washing with distilled water and baked in muffle furnace at 450 °C for 30 min. The dual-chamber MEC reactor (consisting of an anode chamber (28 mL) and a cathode chamber (15 mL)) was constructed to evaluate the potential of *Pseudomonas* sp. E8 to treat simulated AMD. The two chambers of MEC were put together with an anionic exchange membrane (Hangzhou Grion Environmental Technology, Co. Ltd., China) in between. As an important component for our system, the anionic exchange membrane was carried by a positive charge cation group covalently linked to the main chain of polymerization. The cation would not pass through the anion exchange membrane, so as to avoid the pollution of the cathode caused by cation precipitation. The cathode and the electroactive anode of the dual-chamber MEC reactor were connected with an external resistance of 10 Ω. The anode electrodes containing the electroactive biofilm consisting of *Pseudomonas* sp. E8 were enriched in the single-chamber MFC. The cathode was made of carbon cloth (2.5 cm in length × 0.9 cm in width). Duplicate single-chamber MFC reactors or MEC reactors were set up in this study.

### 2.3. Startup and Operation of the MFC Reactor and MEC Reactor

The single-chamber MFCs were inoculated with *Pseudomonas* sp. E8. The 4-fold diluted LB medium (containing peptone 2.5 g/L, yeast extract 1.25 g/L, and NaCl 2.5 g/L) rather than pure chemicals was adopted as the energy source for *Pseudomonas* sp. E8 both in MFC and MEC reactors. MFC reactors were operated in fed-batch mode in a temperature-controlled incubator (30 °C). The medium was replaced once the output voltage of the MFC declined below 20 mV. An adequate amount of cadmium sulfate was dissolved in the diluted bioleaching leachate of chalcopyrite as the simulated AMD in the cathode chamber (pH 1.80) [5]. The catholyte contains (per liter): 50 mg total iron ions, 184.78 mg Cu^2+^, 132.25 mg Cd^2+^. When the removal rate of Cu^2+^ reached above 99.50%, a constant voltage of 1.20 V was applied to the circuit of the MEC by DC power supply in order to recover the Cd^2+^ with low redox potential. The dual-chamber MEC reactor, without the electroactive anode electrodes, was set up as the control.

### 2.4. Measurement and Calculation

The output voltage generated by the MFC was monitored using a voltage collector (ADAM 4117, Advantech, Shenzhen, China) and automatically recorded every 50 s by application software (model 8241, Advantech). The power density and polarization curve were obtained by changing the external resistance (0~51 kΩ) of the MFC after the maximum output voltage of MFCs was stable [21]. Coulombic efficiency of single-chamber MFCs was calculated according to a previous study [22]. The internal resistance of the single-chamber MFCs containing the bioelectroactive anode of this newly isolated strain was measured with electrochemical impedance spectroscopy (EIS) using a potentiostat (Gamry reference 600+ workstation, Philadelphia, Pennsylvania, USA). The EIS measurements were conducted using a three-electrode configuration—the anode of the MFC serving as working electrode and the cathode of the MFC as a counter electrode, and a reference electrode (Ag/AgCl). For each experimental condition, the EIS measurement was conducted in the frequency range from 1000 kHz to 0.01 Hz with an AC amplitude of 5 mV and analyzed by the software of Zview.

The concentration of Cu^2+^ and Cd^2+^ in catholyte were measured by an inductively coupled plasma (ICP) spectrometer (SPECTROBLUE FMS386, AMETEK Materials Analysis Division, Kleve, Germany) after dilution. The concentration of Fe^2+^ and Fe^3+^ in the cathode chamber was determined using the phenanthroline method [23]. The change of pH value of catholyte during the treatment was measured by a pH meter (PHS-3C, Leici, Shanghai). The chemical oxygen demand (COD) removal efficiency (%) of the anolyte was defined as the change of COD concentration divided by the COD concentration in the influent.

### 2.5. Scanning Electron Microscopy (SEM) and X-ray Diffraction (XRD) Analysis

The morphologies of planktonic cells and electroactive biofilm on the anode surface were analyzed by SEM. The planktonic *Pseudomonas* sp. E8 cells in the later exponential phase that were aerobically grown in LB medium were collected by centrifugation at 10,000 g for 5 min. Then, the cells were resuspended and washed in 0.05 M phosphate buffer and fixed with 2.5% (*v*/*v*) glutaraldehyde solution for 3 h. A wisp of anode containing *Pseudomonas* sp. E8 cells was cut and soaked in 2.5% (*v*/*v*) glutaraldehyde solution for 3 h. These fixed samples were dried for 3 h by a vacuum freeze dryer, then coated with platinum and examined with a SEM (JSM-6490LV, JEOL, Tokyo, Japan). The energy dispersive X-ray spectrometry (EDXS; Elect super, EDAX AMETEK, Kleve, Germany) equipped for SEM was used to examine the morphologies and compositions of the deposits on cathode electrodes after the treatment of AMD. The XRD spectra of dried samples were obtained with a Bruker D8 Advance X-ray Diffractometer (D8 Advance, Bruker Corporation, Germany). The data were recorded in the 2θ range of 10 to 80 degrees with a step of 0.02 degrees.

## 3. Results and Discussion

### 3.1. Identification of the Isolated Strain

**Figure 1:**
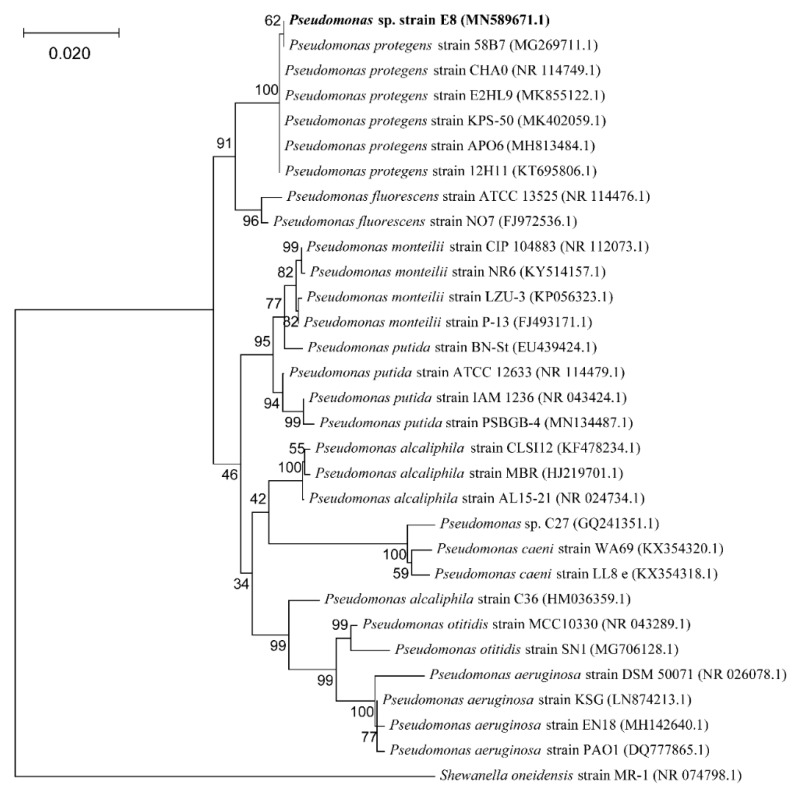
Phylogenetic tree of *Pseudomonas* sp. E8 with the related taxa.

**Figure 2:**
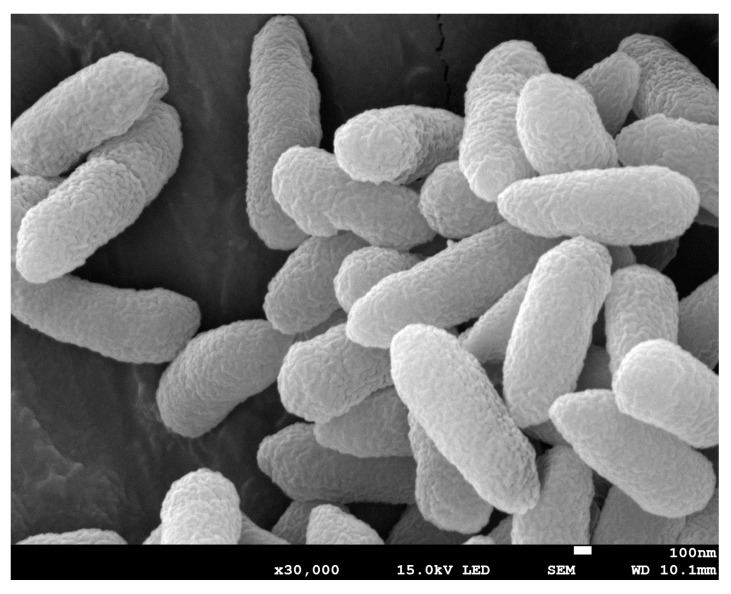
Scanning electron micrographs (SEM) of *Pseudomonas* sp. E8.

**Figure 3:**
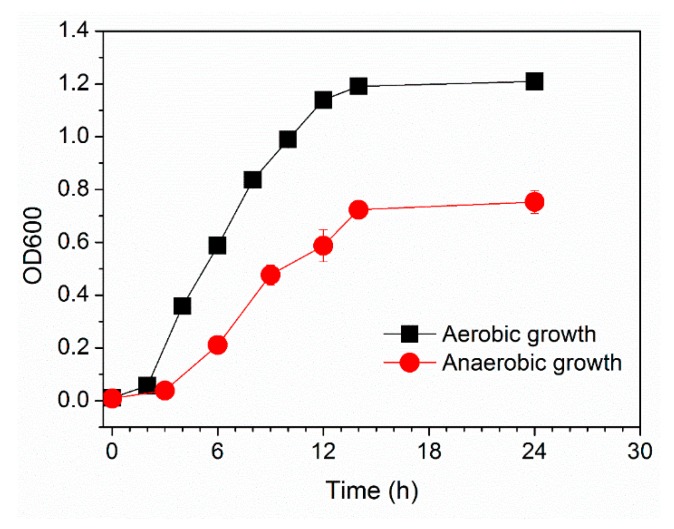
Growth curves of *Pseudomonas* sp. E8 with Luria-Bertani (LB) as substrate under aerobic or anaerobic condition.

The phylogenetic analysis based on the 16S rRNA gene sequencing suggests that this newly isolated exoelectrogenic strain is a member of the genus *Pseudomonas,* as shown in Figure 1. Therefore, this strain was named *Pseudomonas* sp. E8. *Pseudomonas protegens* CHA0 was the nearest neighbor with a high similarity (99.79%) of 16S rRNA sequence. Some exoelectrogenic strains in the *Pseudomonas* genus have been isolated and characterized, such as *P. aeruginosa* PAO1 [24], *P. otitidis* AATB4 [25], *P. monteilii* LZU-3 [26], *P.* sp. C27 [27], *P. alcaliphila* MBR [28], *P. putida* ATCC 49128, and *P. fluorescens* MTCC 2269 [29,30]. *Pseudomonas* sp. E8 is distant from other exoelectrogenic species in the same genus, as shown in Figure 1, which indicates that this strain has different bioelectrochemical activity. The SEM image indicates that the *Pseudomonas* sp. E8 is a rod-shaped bacterium, 0.43–0.47 μm wide and 1.10–1.30 μm long, as shown in Figure 2. *Pseudomonas* sp. E8 is facultative anaerobic, since it grows both aerobically and anaerobically in LB medium, as shown in Figure 3. Much higher cell density was obtained for the aerobically grown culture as compared with that of anaerobically grown culture.

### 3.2. Electrochemical Properties of Pseudomonas sp. E8

**Figure 4:**
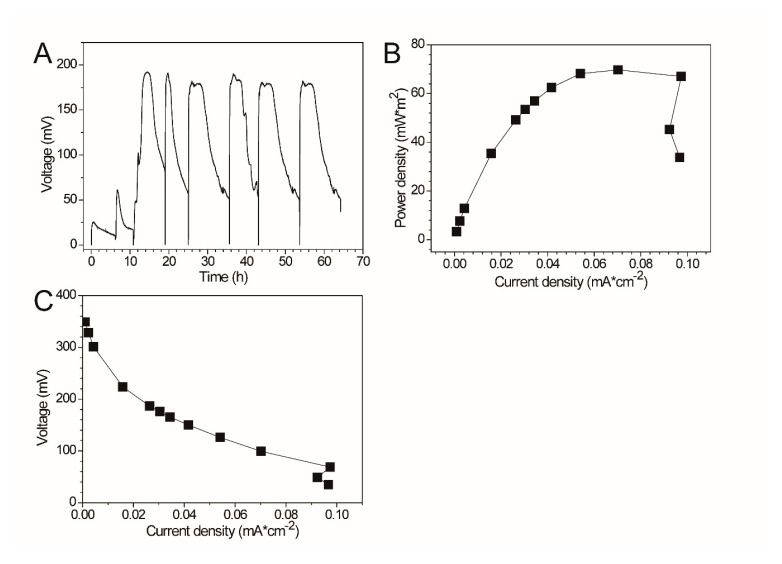
The output voltage (**A**), power density (**B**), and polarization curves (**C**) of a microbial fuel cell (MFC) inoculated with *Pseudomonas* sp. E8.

**Figure 5:**
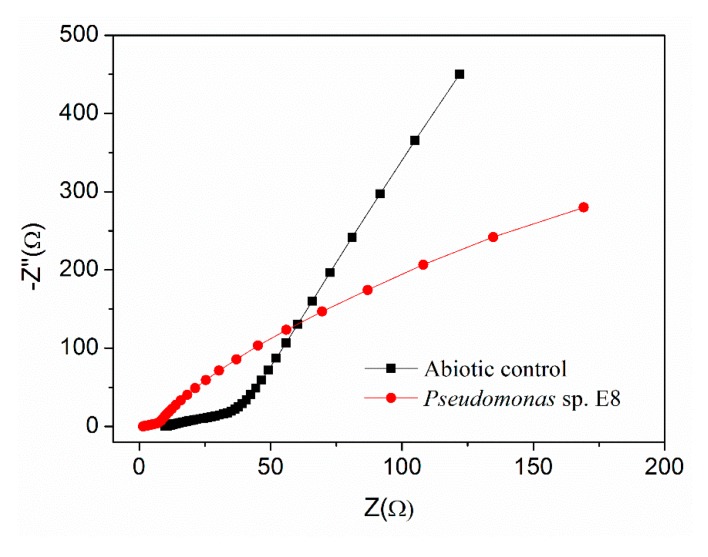
Electrochemical impedance spectroscopy (EIS) analysis of MFC inoculated with *Pseudomonas* sp. E8.

**Figure 6:**
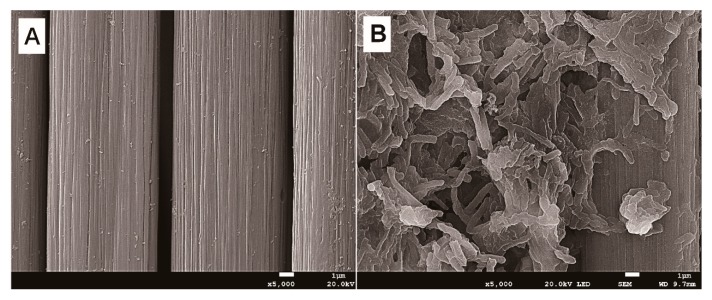
Attachment of *Pseudomonas* sp. E8 on the anode (**A**): abiotic control; (**B**): *Pseudomonas* sp. E8.

The electrochemical properties (e.g., maximum output voltage, power density, polarization curve) of *Pseudomonas* sp. E8 were characterized with fuel cell techniques and compared with those of other exoelectrogenic strains in the genus of *Pseudomonas*. The aerobically grown culture of *Pseudomonas* sp. E8 in LB medium in a shake flask were harvested and inoculated in the single-chamber MFC reactors. The output voltage of the single-chamber MFCs increased to around 25 mV immediately after the inoculation. Thereafter, it started to decrease gradually to 10 mV, as shown in Figure 4A. Then, the single-chamber MFCs were refilled with fresh diluted LB medium. Then, the output voltage rapidly raised to the maximum voltage over 61 mV. After the replacement of the growth medium again, the output voltage rapidly increased to the maximum voltage over 191 mV, and was maintained for around 26 h, then it declined rapidly to 84 mV. Then, the output voltage rapidly increased to the maximum voltage (around 190 mV) again after the removal of planktonic microorganisms by replenishing with fresh diluted LB medium again, which indicates that the current was mainly generated by the attached *Pseudomonas* sp. E8 cells on the surface of anode. The maximum power density of *Pseudomonas* sp. E8 MFC reached 70.40 mW/m^2^ (Figure 4B), which is higher than most other exoelectrogenic strains in the genus of *Pseudomonas* except for *Pseudomonas otitidis* AATB4, as shown in Table 1. The polarization curve shows the output voltage of the MFC containing the bioelectroactive biofilm of *Pseudomonas* sp. E8 under different external resistance, as shown in Figure 4C. The COD depletion for the simulated organic wastewater in the single-chamber MFC inoculated with *Pseudomonas* sp. E8 was 70.63%. The coulombic efficiency of this MFC was 12.70%. The ohmic resistance and charge transfer resistances of these MFCs were obtained by electrochemical impedance spectroscopy (EIS), as shown in Figure 5. A previous study showed that the impedance at the high frequency limit is the ohmic resistance and the diameter of the semicircle is the charge transfer resistance [31]. The ohmic resistance and charge transfer resistances of the MFC without a bioelectrochemical anode were 9.86 Ω and 195.25 Ω, respectively. In contrast, the MFC inoculated with *Pseudomonas* sp. E8 has a much lower ohmic resistance (1.50 Ω) and charge transfer resistances (5.00 Ω). The observation implies that the *Pseudomonas* sp. E8 can greatly reduce the ohmic resistance of the MFC and accelerate the transfer of electrons to the anode. In contrast to the abiotic control, electroactive biofilms that consisted of *Pseudomonas* sp. E8 cells were enriched on the surface of the anode of MFCs when they reached the maximum output voltage as revealed by SEM analysis, as shown in Figure 6.

### 3.3. Sequential Recovery of Cu^2+^ and Cd^2+^ from Simulated AMD

**Figure 7:**
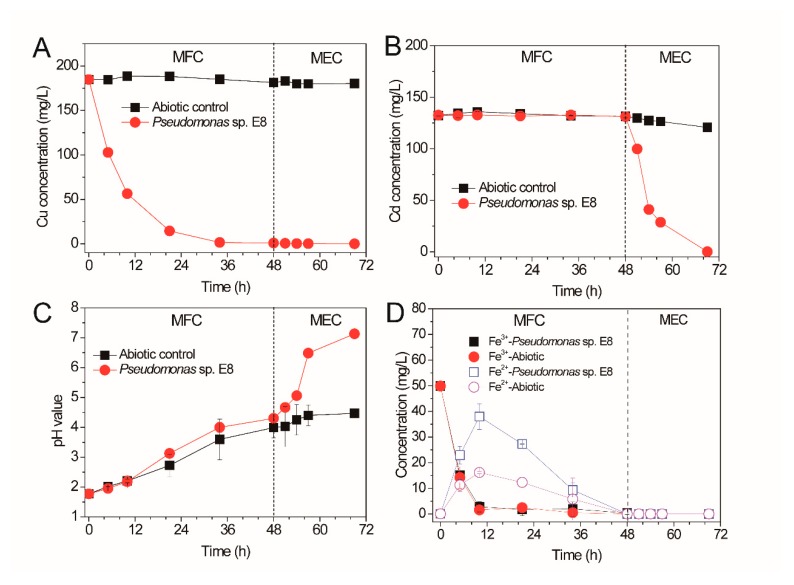
Cu ^2+^ (**A**), Cd^2+^ (**B**) removal rate, pH value (**C**), and iron concentration (**D**) change curve in MFC-microbial electrolysis cell (MEC) cathode chamber.

The Cu^2+^ and Cd^2+^ in simulated AMD were sequentially recovered by a bioelectrochemical system containing the electroactive anode with biofilms of *Pseudomonas* sp. E8 in the modes of MFC and MEC, respectively, as shown in Figure 7. Single-chamber MFCs were dismantled to assemble the dual-chamber MFCs for the treatment of simulated AMD after their maximum output voltage became stable. The Cu^2+^ and Fe^3+^ dissolved in the simulated AMD and acted as electron receptors to accept the electrons transferred from the anode. The Cu^2+^ in the catholyte of MFCs containing the bioelectroactive anode with biofilms of *Pseudomonas* sp. E8 decreased significantly after the initiation of treatment of AMD, as shown in Figure 7A. The concentration of Cu^2+^ in catholyte decreased rapidly from 184.78 to 56.43 mg/L in 10 h, with a high rate of 12.84 mg × L^−1^ × h^−1^. With the further decrease of Cu^2+^ concentration, the copper recovery rate decreased accordingly. A much lower copper recovery rate (2.13 mg × L^-1^ × h^−1^) was observed during the 10th and 34th hour of treatment. According to Wu et al. [33], the copper covered on the carbon cloth of the cathode would decrease the subsequent Cu^2+^ reduction rate. After 48 h of treatment, almost all the Cu^2+^ (99.95 ± 0.09%) in simulated AMD were selectively recovered by MFC, with a low concentration of Cu^2+^ (0.15 mg/L) remaining in catholyte, which meets the standard of China (GB25467-2010). In contrast, the concentration of Cu^2+^ in the catholyte of the MFCs without the bioelectroactive anode were almost unchanged, as shown in Figure 7A. The concentration of Cd^2+^ in the catholyte of all the MFCs were stable because of the large negative redox potential of Cd^2+^. However, the concentration of Cd^2+^ in the catholyte of the MEC containing the bioelectroactive anode of *Pseudomonas* sp. E8 declined significantly to 0.19 mg/L in 21 h, after a constant voltage of 1.20 V was applied by DC power supply, as shown in Figure 7B. Almost all the Cd^2+^ (99.86 ± 0.04%) in simulated AMD was recovered by the microbial electrolysis cell (MEC). However, there was only a slight decrease of the concentration of Cd^2+^ in the catholyte of the abiotic MEC without the bioelectroactive anode, as shown in Figure 7B.

The acidity of the catholyte in the bioelectrochemical system was decreased, as shown in Figure 7C. The pH value of the catholyte in both MFCs with or without the bioelectroactive anode increased after the initiation of treatment. This indicated that the increase of the pH value was likely ascribed to the diffusion of anions from the anolyte across the anion exchange member and reaction with the protons in the catholyte. The pH value of the catholyte in the MECs with the bioelectroactive anode increased significantly to 7.13 ± 0.07 in 21 h, which was probably attributed to the H^+^ reduction occurring on the surface of the cathode. However, only a slight increase of the pH value was observed for the catholyte in the abiotic MECs. The concentration of Fe^3+^ in the catholyte of the MECs with the bioelectroactive anode and the abiotic MECs declined from 50 to 2.81 mg/L and 1.58 mg/L, respectively, as shown in Figure 7D. The decrease of Fe^3+^ in the catholyte should be ascribed to the bioelectrochemical reduction on the cathode of the MECs with the bioelectroactive anode since the Fe^2+^ increased significantly from 0.07 to 37.98 mg/L in the 10th hour after the initiation of treatment. Then, it started to decline to 0 mg/L in the 48th hour. Besides the possible reduction of Fe^3+^ at the cathode, the Fe^3+^ also might be precipitated with a high concentration of SO_4_^2−^ (from the original bioleaching leachate and the CdSO_4_ compound added). It is a surprise that there was also an increase of Fe^2+^ in the catholyte of the abiotic MECs in the beginning 10 h, which is likely ascribed to the cathode potential being near the value of the Fe^2+^/Fe^3+^ redox potential. The decrease of Fe^2+^ in the catholyte should result from the precipitation caused by the elevated pH value after the 24th hour.

**Figure 8:**
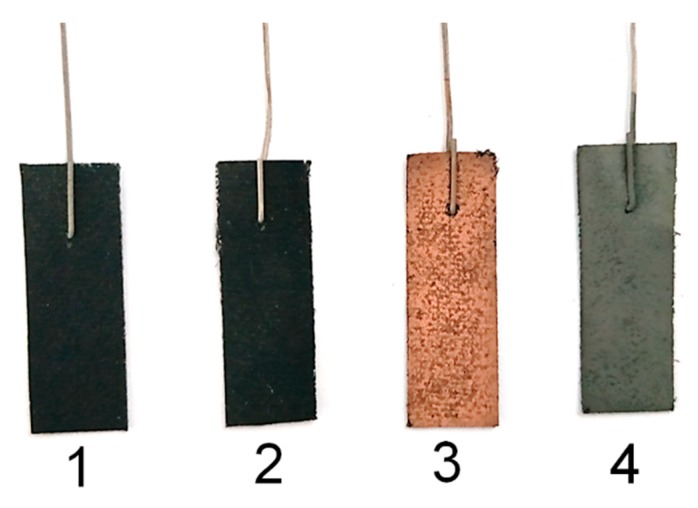
Color change of cathode carbon cloth in different treatment stages (1: before treatment; 2: abiotic; 3: MFC processes; 4: MEC processes).

**Figure 9:**
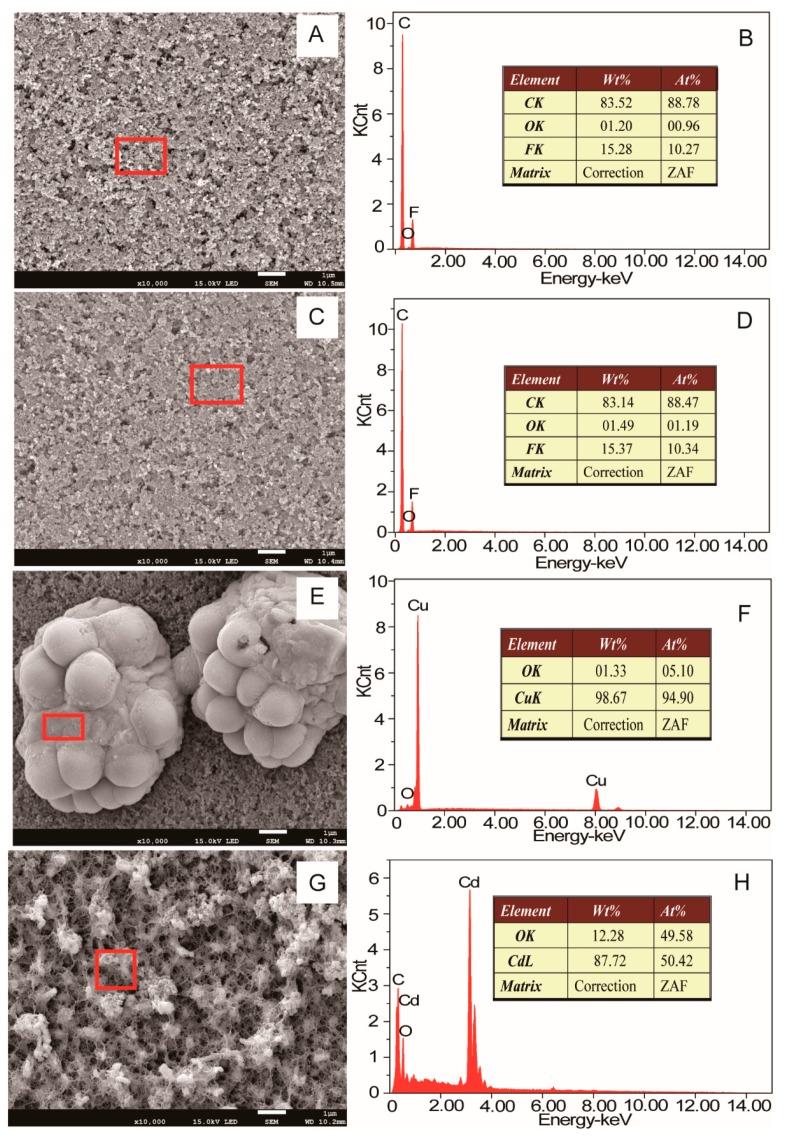
SEM morphology and element composition of the cathode. (**A**,**B**): before treatment; (**C**,**D**): abiotic; (**E**,**F**): MFC; (**G**,**H**): MEC. (The element compositions of regions in red frame were analyzed by EDXS).

**Figure 10:**
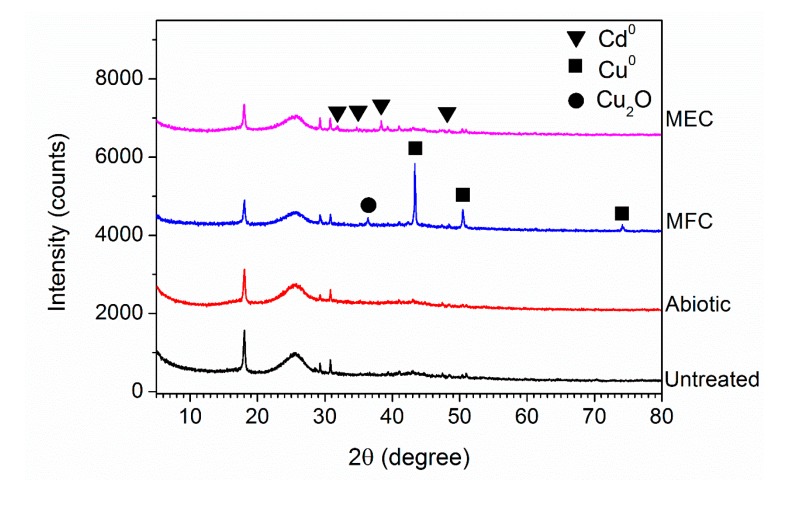
XRD analysis of cathode carbon cloth in different treatment stages.

The color of the cathode carbons of the MFC and MEC were compared with that of the abiotic BES, as shown in Figure 8. The color of the cathode carbon of the MFC containing the bioelectroactive anode turned from black to brown after the treatment of simulated AMD, which indicates the deposit of element Cu on the surface of the cathode. Meanwhile, the grey color of the cathode carbon of the MEC containing the bioelectroactive anode indicates the deposit of element Cd on the surface of the cathode. The morphologies and element composition of the cathode carbon were analyzed with SEM-EDXS, as shown in Figure 9. There were no particles deposited on the surface of the untreated cathode carbon and the cathode carbon of the abiotic BES, as shown in Figure 9A,C. The elements of the cathode carbon mainly consisted of C, O, and F, as shown in Figure 9B,D. Grape-like crystals were deposited on the surface of the cathode of the MFC reactor, which mainly consisted of Cu besides a trace amount of O, as shown in Figure 9E,F. While porous crystals containing elements of Cd, O, and C were formed on the cathode of the MEC reactor, as shown in Figure 9G,H. According to Hu et al., the Cu^2+^ in AMD would undergo two chemical reactions when receiving electrons from the cathode in the MFC reactor [34]. One was that Cu^2+^ received two electrons directly to convert them into elemental crystals, while the other reaction was that Cu^2+^ reacted with water molecules while receiving electrons to form the Cu_2_O crystal. This explained why some oxygen was present on the cathode carbon cloth in the MFC stage, as shown in Figure 9E,F. The XRD patterns of the MFC stage indicated that not only the copper peaks (2θ = 43.3°, 50.4°, and 74.1°) were observed, but also the peak of Cu_2_O (2θ = 36.4°) was detected, as shown in Figure 10. Although a very small amount of Cu_2_O was present, the effect on the quality of the recovered copper metal was negligible. Similarly, according to Zhang et al. [35], cadmium removal in the microbial electrolysis cell was realized by cathode reduction or electrodeposition. However, this was not the only cadmium removal mechanism. Cadmium could also be removed by precipitation as cadmium hydroxide or precipitate as cadmium carbonate [36]. In this study, the qualitative element analysis of the cathode carbon cloth in the MEC stage showed that there are three elements, C, O, and Cd, among which cadmium had two valence states. This suggested that the materials attached to the cathode carbon cloth in the MEC stage may be composed of cadmium and cadmium carbonate. According to the XRD results, the characteristic peak of cadmium elemental was present when 2θ was 38.3°, while the characteristic peak of cadmium carbonate was present when 2θ was 31.2°. However, based on the above results, the polymer attached to the cathode carbon cloth was mainly composed of cadmium. Collectively, *Pseudomonas* sp. E8 has the potential to simultaneously treat organic wastewater and recover Cu^2+^ and Cd^2+^ from AMD by the way of BES.

The expression of functional genes in either a single strain or a microbial consortium were altered by various physicochemical parameters [5,37,38]. Therefore, it is necessary to identify the important genes involved in the electron transfer for electricity generation of this novel exoelectrogen by comparative transcriptomic analyses of the attached cells on the surface of the anode and the planktonic cells in the MFC in the near future. The extracellular polymeric substances (EPS) are important for the function roles of single microorganism and consortium [39,40]. Characterization the compositions and redox properties of the EPS of *Pseudomonas* sp. E8 attached on anodic surface will provide novel insights into the function role of EPS in mediating electron transfer.

## 4. Conclusions

A novel exoelectrogenic strain, *Pseudomonas* sp. E8, was first isolated in this study. Phylogenetic analysis shows that *Pseudomonas* sp. E8 is distant from other exoelectrogenic species in the same genus. The bioelectrochemical activity of *Pseudomonas* sp. E8 was evaluated by microbial fuel technology. Maximum power density of *Pseudomonas* sp. E8 is much higher than most other exoelectrogenic *Pseudomonas* species. The electroactive biofilms enriched on the surface of the anode, rather than the planktonic culture, play the pivotal role in the generation of electricity. The Cu^2+^ and Cd^2+^ in simulated AMD were sequentially recovered by the bioelectrochemical system containing the electroactive anode with biofilms of *Pseudomonas* sp. E8 in the modes of MFC and MEC, respectively. The Cu^2+^ and Cd^2+^ in simulated AMD were recovered by bioelectrochemical reduction to Cu^0^ and Cu_2_O, or Cd^0^, respectively, on the surface of the anode. Further research works are needed to assess the technical feasibility of the bioelectrochemical system containing *Pseudomonas* sp. E8 to treat AMD, such as scaling-up the reactor and running in continuous mode. In addition, identification of the important genes involved in the electron transfer for electricity generation of this novel exoelectrogen by comparative transcriptomic analyses are needed. Collectively, data indicate that *Pseudomonas* sp. E8 has great potential for AMD treatment and metal recovery.

## Figures and Tables

**Table 1:** Comparison of the bioelectroactive species in the *Pseudomonas* genus.

Name of Bacterial Species	Device Type	Anode Material	Cathode Material	Exchange Membrane	Energy Substrate	Coulombic Efficiency	Maximum Power Density	Reference
*Pseudomonas aeruginosa* PAO1	single-chamber MFC (180 mL)	Pt-loaded carbon cloth (7 cm^2^)	Pt-loaded carbon cloth (7 cm^2^)	\	glucose	\	42.53 μW/cm^2^	[24]
*Pseudomonas putida* ATCC 49128	single-chamber MFC (40 mL)	a carbon fiber brush (area, 17.7 cm^2^)	carbonized 50 × 50 mesh stainless steel	\	bilge water	27.20%	0.04 mW/m^2^	[29]
*Pseudomonas fluorescens* MTCC 2269	single-chamber MFC (180 mL)	graphite block	treated carbon cloth (9 cm^2^)	\	glucose	\	83.04 ± 4.0 μW/m^2^	[30]
*Pseudomonas otitidis* AATB4	dual-chamber MFC (each chamber 250 mL)	graphite electrode	graphite electrode	proton exchange membrane	septic tank wastewater	\	218 mW/m^2^	[25]
*Pseudomonas monteilii* LZU-3	dual-chamber MFC (each chamber 240 mL)	carbon felt (16 cm^2^)	carbon felt (16 cm^2^)	proton exchange membrane	p-nitrophenol	\	31 mW/m^2^	[26]
*Pseudomonas* sp. C27	dual-chamber MFC (each chamber 78 mL)	carbon felt (area, 6 cm^2^)	carbon cloth (area, 9 cm^2^)	cation exchange membrane	sulfide	25.60%	40 mW/m^2^	[32]
*Pseudomonas alcaliphila* MBR	dual-chamber MFC (each chamber 245 mL)	carbon felt (30 cm^2^)	carbon felt (30 cm^2^)	cation exchange membrane	sodium citrate	\	\	[28]
*Pseudomonas* sp. E8	single-chamber MFC (28 mL)	carbon brush	carbon cloth (area, 7 cm^2^)	\	LB	12.70%	70.4 mW/m^2^	This study
